# Dr. William Hyser

**Published:** 1909-05

**Authors:** 


Dr. William Hyser, of Plainfield, Mich., died at his home March 20, 1909, aged 82 years. He graduated at the University of Buffalo in 1850.

